# A Case of *Helicobacter cinaedi* Bacteraemia in a Previously Healthy Person with Cellulitis

**DOI:** 10.2174/1874285800802010029

**Published:** 2008-04-03

**Authors:** Helle Holst, Keld Andresen, Jens Blom, Niels Højlyng, Michael Kemp, Karen Angeliki Krogfelt, Jens Jørgen Christensen

**Affiliations:** 1 Department of Bacteriology, Mycology and Parasitology; 2 Department of Virology, Statens Serum Institut; 3 Infectious Diseases Unit, Medical Department, Roskilde University hospital; Copenhagen, Denmark

**Keywords:** Helicobacter cinaedi, heterosexual, xtra-intestinal setting, infection, PCR

## Abstract

*Helicobacter cinaedi* is an infrequent, but well recognized cause of gastroenteritis in immunosuppressed patients. Here we report a case of an extra-intestinal infection in a previous healthy 61-year old heterosexual male. Focus for the infection was most likely cellulitis on the lower right leg. The bacterium was cultured from blood twice within one week. Electron microscopy of the isolate visualized bipolar flagella. Partial DNA sequencing of the 16S rRNA gene and phenotypic characterization of the isolate established the species diagnosis. The patient was treated with rifampicin. After end of treatment blood cultures were negative and the cellulitis had disappeared.

## INTRODUCTION

Helicobacter cinaedi belongs to the enterohepatic helicobacters. They inhabit the lower gastrointestinal tract of mammals and birds and have been implicated as cause of human gastroenteritis, particularly in immunocompromised individuals [1]. In contrast to Helicobacter pylori which very rarely invade the bloodstream, the enterohepatic helicobacters may do so. Of these, H. cinaedi is the most frequently reported bloodstream invading organism, especially in immunocompromised hosts and particularly in men infected with HIV [3]. Only few cases of bacteraemia with H. cinaedi have been observed in heterosexual immunocompetent men [3]. In the case presented here, the patient suffered from bacteraemia with H. cinaedi and had a large area with erysipelas-like infection on his lower right leg – a characteristic clinical picture seen previously in relation to blood-stream infection with H. cinaedi [3]. The initial identification of H. cinaedi was established by a culture independent method; i.e. PCR using 16S rDNA universal bacterial primers and subsequent DNA sequence analysis of the PCR product [4]. Also given the problems associated with conventional phenotypic identification of organisms like H. cinaedi, sequence analysis of rRNA genes (in particular 16S) is increasingly used as an alternative approach for identification of unspecified isolates. In our case supplementary electron microscopy was carried out. Due to the slow growth and fastidious requirements of this organism, H. cinaedi bacteraemia may currently be under-recognized.

## CASE PRESENTATION

### Case Story

A 61-year old heterosexual male was admitted to hospital with an infection on his lower right leg. Before admission he was treated empirically with oral beta-lactam antibiotics for a few days and then changed to dicloxacillin for three days

by his general practitioner. On admission he had fever, chills and a leukocyte count of 15,9x10^9^ /l. and a C-reactive protein concentration of 77mg/l. The condition was at first considered to be erysipelas and treated with intravenous penicillin 2mill. IU x 4. The patient was discharged after three days of intravenous treatment and continued with oral penicillin treatment for another week. He was readmitted, however, two days later because the cellulitis had progressed. Blood cultures were taken at both admissions and gave by both episodes growth of motile spiral-shaped Gram-negative rods after 5 days incubation. At readmission PCR/sequencing on the first blood-culture sample indicated presence of *H. cinaedi* and treatment was then changed to peroral rifampicin 300 mg once daily for 2 weeks. At the end of treatment no helicobacters were detected in blood cultures and the skin alterations had disappeared. Fecal specimens were not available. Antibodies reacting against *H. pylori* were detectable in serum. No recurrences were noted at 5 months follow-up. Source of infection is unknown; no contact to domestic animals was reported. Exposure to mice excrements was reported, while cleaning the attic of his house. Focus for the bacteraemia was most likely cellulitis on the lower right leg.

### Microbiology

**Fig. (1):**
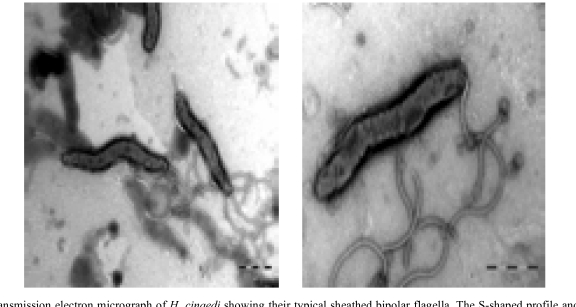
Transmission electron micrograph of *H. cinaedi* showing their typical sheathed bipolar flagella. The S-shaped profile and terminal bulbs are also illustrated.

Bacterial isolates were recovered from blood samples using the Bactec 9240 system (Becton Dickinson).One set of blood cultures consist of two Bactec Plus aerobic/F bottles and one Bactec Plus anaerobic/Bottle. In both sets of blood cultures aerobic blood cultures (2 bottles out of 4) became positive after 5 days of incubation. Light microscopy of the blood culture fluid identified a spiral-shaped, motile, and Gram-negative rod.The organism failed to grow on 5% horse blood agar, but grew successfully at microaerophilic conditions at 37°C on horse blood agar enriched with 5% yeast. Electron microcopy visualized bipolar sheathed flagella (Fig. **1**). Electron microscopy was carried out using a FEI Morgani D268 electron microscope at 80 kW. Bacteria grown for two days were harvested from blood agar plates and gently resuspended in distillated water. Bacterial ultra structure was examined by the negative staining technique using 1.5% phosphotungstic acid.

Demonstration of bacterial DNA and partial DNA sequencing of the 16S rRNA was performed on blood-culture fluid and on the isolate. DNA was released using the Qiagen extraction kit or heating isolated bacteria at 95^o^ C for five minutes. For demonstrating presence of bacterial DNA the universal primers BSF-8/BSR-534 and BSF-8/BSR-1409 were used as previously described [4]. The data obtained were compared to deposited sequences in the NCBI database using the BLAST search engine. Blast files were evaluated with respect to %/ number of identities, score (bits) and E-values for the best and the next best matches. When using the primers BSF-8/BSR-534, BLAST examination of the obtained sequence showed a high degree of base similarity with *H. cinaedi *(Identities 487/487, 100%, Maxscore of 965 bits) and *Flexispira rappini *(Identities 486/487, 99%, Maxscore of 957 bits). A subsequent BLAST examination was performed whereby 87% of the 16S rRNA genome was sequenced (primers BSF-8/BSR-1409) confirming *H. cinaedi* (Identities 1255/1256, 99%, Maxscore of 2314 bits) to be prior to *F. rappin*i (Identities 1245/1256, 99%, Maxscore of 2259 bits). Further, *H. cinaedi* appeared 18 times as best taxon match before *F. rappini* appeared in the BLAST search. The 16SrRNA gene sequence has been deposited in the Gen Bank Database under accession number EU589201.

To unambiguously confirm the identification of the isolate based on phenotypic distinctions between *H. cinaedi and Flexispira species*, differentiating growth capabilities and biochemical features were set up for 4 species including the patient isolate; the following culture collection strains from Culture Collection of University of Gothenburg, Sweden (CCUG) were used:* H cinaedi (CCUG 18818), H. fennelie (CCUG 18820) and Flexispira 8 *(CCUG 23435).

Characteristics used to differentiate, as recommended by Fox and Megraud [1] gave the following results for the blood isolate and *H. cinaedi* (CCUG 18818): Catalase-positive, nitrate reductase-positive, but alkaline phosphatise, urease, indoxyl acetatehydrolysis, and gamma-glutamyltransferase negative. The isolate did not grow in air at 37˚C, and grew slowly at 37˚C at microaerophilic conditions, but not at 42˚C and 25˚C. The isolate was susceptible to nalidixic acid and resistant to cephalotin using disk diffusion technique. For treatment guidance the isolate was tested and found sensitive to rifampicin (E-test of 0.064mg/L). *Flexispira 8* and *H. fennelliae* could be separated on the basis of different reactions for, respectively, catalase, urease- plus gamma-glutamyl transferase production and susceptibility to cephalotin.

## COMMENTS

*Helicobacter species* have been isolated from the gastrointestinal and hepatobiliary tracts of mammals and birds. *H. cinaedi*, has been isolated from humans, hamsters, macaques, foxes, rats, and dogs. In relation to that no known animal exposition was noted for this patient. In humans, *H. cinaedi* is most commonly isolated from homosexual men (“cinaedi” is latin for “homosexuals”) infected with HIV, but can also occur in other immunocompromised patients or, occasionally, in healthy hosts [5]. This patient is an example of the latter as the patient did not belong to any of the mentioned risk groups. Enterohepatic helicobacters can cause diarrhea and disseminated infection with positive blood cultures. Nearly all patients have fever and a relatively mild , subacute course. Leukocytosis and thrombocytopenia occur variably. Interestingly, colitis is typically not seen in patients with H. cinaedi bacteremia. However, a chronic focal (may be multifocal) cellulitis, usually of the lower extremities, is very common [3,6]. A presentation identical to the one seen in this case.

Antimicrobial agents that have been found active against *H. cinaedi* includes doxycycline, rifampicin, ceftriaxone and imipenem [3,7]. Strains may be resistant to erythromycin and ciprofloxacin as *in vitro* results have been mixed [3,2]. Antimicrobial therapy should be continued for a minimum of 2 weeks and perhaps longer for patients with bacteremia. In view of several reports of recurrent infection [6], additional blood cultures after completion of therapy should be considered.

Molecular methods have proven beneficial in cases of fastidious isolates with special growth needs and where antibiotic treatment has preceded specimen sampling. In our case microscopy revealed Gram-negative rods, which however did not grow by standard procedures. It has recently been reported that use of enriched blood culture media as the Bactec Peds Plus (Becton Dickinson) provides better growth with fastidious organisms as *Helicobacter canis* [8] The use of 16S rRNA gene sequencing of extracted DNA from the blood culture fluid pointed on *H. cinaedi* as the etiology and optimizing growth conditions according to this information resulted in bacterial growth. This gave the opportunity to compare molecular and phenotypic results and obtain a definite diagnosis as both methods supplemented each other. Solely based on molecular results (16S rRNA gene sequencing) it would not be possible to reach a secure species designation, which has been observed previously within this group of bacteria as species by this method are very closely related. Likewise, a relatively biochemically inert bacteria as *H. cinaedi* may be extremely difficult to identify on only phenotypic reactions.

Thus, the combination of molecular results with phenotypic characteristics, including a demonstration of the bipolar sheathed flagella, confirmed the species designation. A valuable hint was given by the molecular results thereby making it possible to grow the isolate for further characterization.
